# The predictive prognostic value of serum CEA, CA153, HE4 combined with a variety of immune inflammatory indicators in breast cancer

**DOI:** 10.4314/ahs.v24i4.29

**Published:** 2024-12

**Authors:** Ping Zou, Ke Li, Yiting Qian, Tingting Wu, Yue Qian

**Affiliations:** 1 Department of General Surgery, The First Affiliated Hospital of Soochow University, China; 2 Department of Plastic and Burn Surgery, The First Affiliated Hospital of Soochow University, China; 3 Department of Gastroenterology, The First Affiliated Hospital of Soochow University, China; 4 Department of Dermatology, The First Affiliated Hospital of Soochow University. China

**Keywords:** Breast cancer, CA153, CEA, HE4, immune inflammatory indicators, predictive, prognostic

## Abstract

**Objective:**

To investigate the prognostic value of serum carcinoembryonic antigen (CEA), carbohydrate antigen (CA153), and human epididymal 4 (HE4) combined with a variety of immune inflammatory indicators in breast cancer.

**Methods:**

A sample size of 112 breast cancer patients was selected and all patients underwent surgical treatment. The three-year prognosis of the patients was observed. Patients with poor prognoses were included in the poor prognoses group and the patients with good prognosis were included in the good prognosis group. The levels of serum CEA, CA153, HE4, and related immune inflammatory indicators (including Mon, Lym, Neu, Plt, NLR, and SII) were analysed. The predictive value of serum CEA, CA153, HE4, and immune inflammatory indicators on the prognosis of breast cancer was analysed by Logistic regression and ROC curve method. The area under the ROC curve (AUC) was used to evaluate the predictive efficiency. α=0.05 was used as the test standard.

**Results:**

Compared with the good prognosis group, the CEA, CA153, HE4, Mon, Neu, Plt, and SII of the poor prognosis group were higher, all P<0.05. Logistic regression analysis showed that CEA, CA153, HE4, Neu, and Plt were effective indicators for predicting the poor prognosis of breast cancer. The AUC of CEA, CA153, HE4, Neu, and Plt in predicting the prognosis of breast cancer was 0.929, which was higher than the AUC of a single prediction.

**Conclusion:**

Serum CEA, CA153, HE4 combined with Neu and Plt had predictive value for the prognosis of breast cancer.

## Introduction

Breast cancer accounts for 24.2% of all female malignant tumors and ranks second among the causes of female cancer death. 1 The incidence of breast cancer ranks first among female malignant tumors and has a trend of the increasing year by year. Surgery is the treatment of choice for breast cancer. However, a large number of clinical practices have found that the incidence of postoperative recurrence or metastasis in breast cancer patients is high. 2 And recurrence and metastasis are important factors causing the death of patients. 3 Prediction of the prognosis of breast cancer (recurrence or metastasis) can guide clinical treatment and intervention. At present, there is still a lack of a timely and effective prediction method for breast cancer.

Currently, in the postoperative monitoring of breast cancer, laboratory examination indexes have little clinical value, and imaging techniques are difficult to detect small lesions. A large number of tumor markers in serum can be diagnosed in the early stage of tumor occurrence.^4^ It had been confirmed that the occurrence and development of tumors were related to a variety of biological factors. 5 Studies had shown that serum carcinoembryonic antigen (CEA) 6, carbohydrate antigen (CA153) 7, human epididymal 4 (HE4)^8^, and immune inflammatory indicators 9 were related to the occurrence of breast cancer. Therefore, it is speculated that the combined detection of serum CEA, CA153, HE4, and immune inflammatory indicators may have significance in the detection of breast cancer prognosis (recurrence or metastasis).

After a large amount of literature review, we found out that, there were almost no studies on the combined detection of serum CEA, CA153, HE4, and immunoinflammatory indicators in the prediction of breast cancer prognosis. Previous studies were mostly devoted to exploring the role of one or two of the four indicators of CEA, CA153, HE4, and immunoinflammatory indicators in the diagnosis of breast cancer. Rather than exploring their role in predicting breast cancer outcomes. Therefore, we speculate that there may be new breakthroughs in this research to guide clinical treatment decisions. At present, there is lack of a stable and reliable method to predict the prognosis of breast cancer. Therefore, by observing CEA, CA153, HE4 and immune inflammation indicators of breast cancer patients in our hospital, this study analysed the value of the above indicators combined in predicting the prognosis of breast cancer and the results were reported as follows.

## Materials and Methods

### Patients with source

A sample size of 112 breast cancer patients diagnosed and treated in our hospital from June 2016 to May 2019 was selected. The included patients should meet the following conditions: According to relevant evaluation criteria, the postoperative pathological diagnosis of breast cancer; did not receive any antitumor therapy before diagnosis, and underwent surgery after diagnosis; Preoperative whole body bone scan and puncture were performed to confirm no recurrence or metastasis; Clinical data and detection results of CEA, CA153, HE4, and immunoinflammatory indicators were complete; The patients were informed of this study and agreed to refer to their case data. Patients with the following conditions were excluded: Complicated with serious cardiovascular and cerebrovascular diseases; Complicated with autoimmune diseases; Malignant tumors other than breast cancer; Male breast cancer; Death from breast cancer during follow-up. This study has been approved by the Medical Ethics Committee of the First Affiliated Hospital of Soochow University in China.

### Index detection

Tumor markers: 5 mL upper arm venous blood was collected preoperatively at 3000 r/min, centrifuged for 10 min, and serum was isolated and stored in a refrigerator at -20°C for examination. The levels of CEA, CA153, and HE4 were measured by electrochemiluminescence (Roche diagnostic kit), and the measurements were performed strictly according to the instructions. Reference range: CEA: 0–5 ng/mL; CA153: 0–28.5 U/mL; HE4: 0–70 pmol/L before menopause, 0–140 pmol/L after menopause. Indicators of immune inflammation: The thickness of specimens was 5 µm before operation and HE staining was used for identifying the areas with dense inflammatory infiltration under a low-power microscope. The monocyte count (Mon), lymphocyte count (Lym), neutral particle count (Neu), and platelet count (Plt) of any 10 high-power fields (HPF) were counted by two pathologists using a 400× high-power microscope field. NLR (Neu/Lym) and Systemic immune inflammation (SII) index were calculated as Neu×Plt/Lym 10.

### Follow-up and grouping

The main follow-up method in this study was telephone follow-up every 3–6 months. All patients were followed up until June 30th, 2022. Diagnosis of postoperative recurrence and metastasis of breast cancer: Postoperative review, including breast B-type ultrasound, chest X-ray film, serum breast tumor marker examination, etc. Recurrence included recurrence in the ipsilateral breast, recurrence in the operation area, local metastasis, etc., which was confirmed by surgical mass resection or biopsy pathology 11. Recurrence or metastasis of breast cancer during follow-up was considered as the termination event, occurrence of recurrence or metastasis was considered as poor prognosis (included in the poor prognosis group), and no recurrence or metastasis was considered as good prognosis (included in the good prognosis group).

### Statistical method

The data of this study were processed by SPSS 23.0 software, the measurement data were represented by “^-^x±s”, and the comparison between the two groups was analysed by t-test. The counting data were represented by n (%), and the X^2^ test was conducted. When 1≤ theoretical frequency <5, the chi-square was the correct value, and when the theoretical frequency < 1, the chi-square was the exact value. The predictive value of serum CEA, CA153, HE4, and immune inflammatory indicators on the prognosis of breast cancer was analysed by Logistic regression and ROC curve method. The area under the ROC curve (AUC) was used to evaluate the predictive efficiency. a=0.05 was used as the test standard.

## Results

### Baseline information

The age, BMI, surgical method, and pathological stage of patients in the poor prognosis group and the good prognosis group were compared, all P>0.05. See Table 1.

**Table 1:** Baseline information

Group	Age(x̅±s,years)	BMI(x̅±s,kg/m^2^)	Surgical method [n(%)]				Pathological stage	

			radical mastectomy	modified radical surgery	breast conserving surgery	simple excision of pterygium	stage III	Stage IV
Poor prognosis group(n=75)	60.12±10.23	23.41±1.33	38(50.67)	22(29.33)	10(13.33)	5(6.67)	36(48.00)	39(52.00)
Good prognosis group(n=37)	59.86±11.04	23.12±1.54	23(62.16)	10(27.03)	3(8.11)	1(2.70)	21(56.76)	16(43.24)
t/χ^2^	0.123	1.029	2.074				0.760	
*P*	0.902	0.306	0.557				0.383	

### Tumor marker

**Figure 1:**
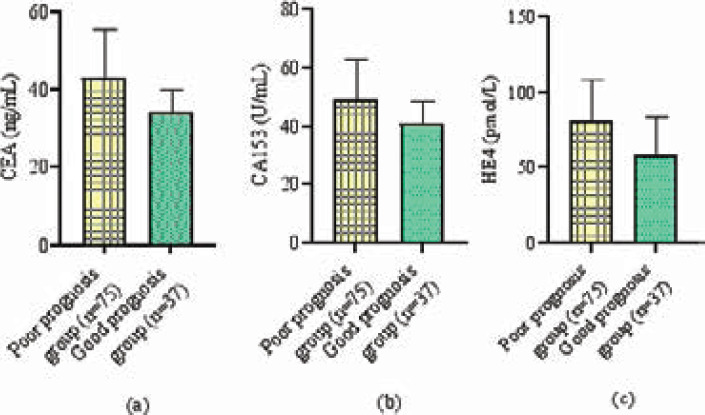
Tumor marker levels

Compared with the good prognosis group, the CEA, CA153, and HE4 of the poor prognosis group were higher (P<0.05). As shown in Table 2 and Figure 1.

**Table 2:** Tumor marker levels (x̅±s)

Group	CEA (ng/mL)	CA153(U/mL)	HE4(pmol/L)
Poor prognosis group(n=75)	43.26±12.25	49.37±13.44	81.38±27.02
Good prognosis group(n=37)	34.10±5.90	40.98±7.59	58.36±25.20
t	4.302	3.525	4.334
*P*	<0.001	<0.001	<0.001

Compared with the good prognosis group, the CEA, CA153, and HE4 of the poor prognosis group were higher (P<0.05).

### Immune inflammatory indexes

**Figure 2:**
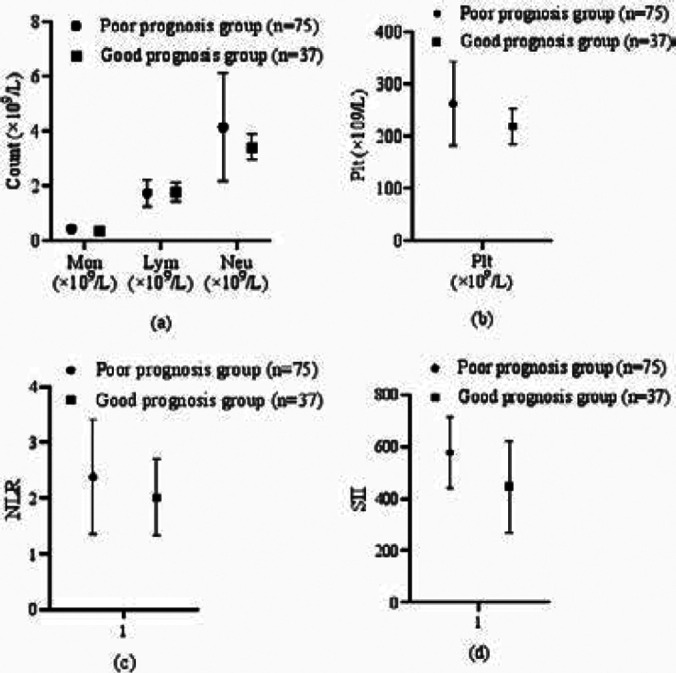
Immune inflammatory indexes levels

In comparison with the good prognosis group, the Mon, Neu, Plt, and SII of the poor prognosis group were higher (P<0.05). See Table 3 and Figure 2.

**Table 3:** Immune inflammatory indexes levels (x̅+s)

Indexes	Poor prognosis group(n=75)	Good prognosis group(n=37)	t	*P*
Mon(×10^9^/L)	0.45±0.10	0.37±0.10	3.982	<0.001
Lym(×10^9^/L)	1.76±0.47	1.79±0.35	0.344	0.732
Neu(×10^9^/L)	4.18±1.98	3.45±0.46	2.209	0.029
Plt(×10^9^/L)	264.67±81.28	220.09±33.82	3.197	0.002
NLR	2.40±1.03	2.03±0.68	1.980	0.050
SII	579.85±135.75	448.58±173.96	4.375	<0.001

In comparison with the good prognosis group, the Mon, Neu, Plt, and SII of the poor prognosis group were higher (P<0.05).

### Logistic regression

The prognosis of breast cancer patients was taken as the dependent variable (0=good prognosis, 1=poor prognosis), and CEA, CA153, HE4, Mon, Lym, Neu, Plt, NLR, and SII were taken as covariates to be included in the Logistic regression analysis. CEA, CA153, HE4, Neu, and Plt were effective indicators for predicting the poor prognosis of breast cancer. See Table 4.

**Table 4:** Logistic regression analysis results

Variable	*β*	*S.E*	*P*	*OR*	95% *CI*	

					lower limit	upper limit
CEA	0.124	0.038	0.001	1.132	1.051	1.219
CA153	0.079	0.026	0.002	1.083	1.029	1.139
HE4	0.039	0.014	0.004	1.04	1.013	1.068
Neu	0.966	0.327	0.003	2.628	1.385	4.986
Plt	0.028	0.008	0.001	1.028	1.012	1.044

### Prognostic efficacy of CEA, CA153, HE4, Neu, and Plt in breast cancer

**Figure 3:**
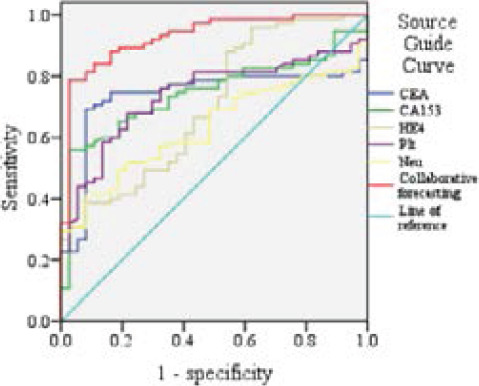
ROC curves of CEA, CA153, HE4, Neu, and Plt in predicting the prognosis of breast cancer

CEA, CA153, HE4, Neu, and Plt were used as predictors to draw the ROC curve of CEA, CA153, HE4, Neu, and Plt for the prognosis of breast cancer. The combined AUC of CEA, CA153, HE4, Neu, and Plt was 0.929, which was higher than the AUC of a single prediction. See Table 5 and Figure 3.

**Table 5:** Prognostic efficacy of CEA, CA153, HE4, Neu, and Plt in breast cancer

Variable	AUC	*S. E*	*P*	95% *CI*		Optimal cut-off value	Sensitivity (%)	Specificity (%)

				lower limit	upper limit			
CEA	0.736	0.050	<0.001	0.638	0.834	35.06	69.3	91.9
CA153	0.745	0.046	<0.001	0.654	0.836	50.97	56.0	97.3
HE4	0.704	0.052	<0.001	0.602	0.806	52.66	88.0	45.9
Neu	0.632	0.051	0.024	0.531	0.732	4.07	41.3	91.9
Plt	0.738	0.047	<0.001	0.645	0.830	249.06	68.0	78.4
Collaborative forecasting	0.929	0.025	<0.001	0.879	0.978	0.76	78.7	97.3

The combined AUC of CEA, CA153, HE4, Neu, and Plt for the prognosis of breast cancer was 0.929, which was higher than the AUC of a single prediction.

## Discussion

The incidence of breast cancer is high, which greatly endangers the health of the public, especially women. Most literature reports indicate that the incidence of postoperative recurrence and metastasis of breast cancer was high, with recurrence reaching more than 35% 12.

Accurate monitoring of breast cancer recurrence and metastasis is of positive significance to improve the benefit of treatment. In recent years, molecular biology has made phased progress and a variety of tumor markers have been discovered, creating the possibility of tumor tissue monitoring. In addition, more and more studies had shown that inflammation may affect the occurrence and development of cancer by promoting tumor survival/proliferation/migration, promoting angiogenesis, and inhibiting anti-tumor immunity 13.

Therefore, it is necessary to explore the predictive role of related tumor markers and immune inflammatory indicators in breast cancer recurrence and metastasis.

This study analysed the predictive value of CEA, CA153, and HE4 combined with various immune inflammatory indicators (including Mon, Lym, Neu, Plt, NLR, and SII) in the prognosis of breast cancer. By analysing the clinical data of breast cancer patients in a single center, tumor markers and immune inflammatory indicators mentioned in previous studies that may be related to the prognosis of breast cancer were included in the study for the first time and the possible predictive value of these indicators was preliminarily verified. Then Logistic regression analysis was used for evaluating their potential value in the prognosis of breast cancer. This study found out that patients with high CEA, CA153, HE4, Neu, ad Plt were more likely to have a poor prognosis. These indicators may play an important role in the process of postoperative recurrence and metastasis of breast cancer. In-depth analysis showed that CEA, CA153, HE4, Neu, and Plt were effective indicators for predicting the prognosis of breast cancer and the combined AUC of each indicator was 0.929, which was higher than the AUC of a single prediction, indicating that the combined detection had higher predictive value.

In the diagnosis of breast cancer, CEA and CA153 were common serum markers in clinical practice. This study showed that high CEA and CA153 were associated with poor prognosis of breast cancer, which was consistent with relevant research results 14.

CEA existed in colon tissue and was in a state of low expression in the normal body which was a non-specific tumor marker and was often used in combination with other indicators in tumor diagnosis 15.

CA153 was a recognized breast cancer-related antigen, which was highly expressed in tumor cells, and its positive rate was significantly increased in patients with metastatic lesions 16.

Some studies had pointed out that elevated CEA levels indicated the risk of systemic metastatic disease in patients 17.

Other studies had pointed out that the ratio of CEA to neutrophil and lymphocyte (NLR) could well evaluate the prognosis of colorectal cancer 18.

CA153 was also a key indicator for predicting early breast cancer, and combined detection could improve the sensitivity of diagnosis 19.

In previous studies and analyses, it was believed that the content of tumor markers increased significantly in breast cancer patients with recurrence and metastasis 20.

Some studies had also pointed out that the expression level of tumor markers was related to tumor burden, and serum CEA was significantly increased in patients with organ metastasis, and the metastasis range was wider and the content was higher. In early breast cancer, too high CEA indicates the risk of postoperative recurrence and metastasis. As an emerging tumor marker, HE4 had a high expression level in breast cancer, ovarian cancer, and endometrial cancer, and had a high predictive value for disease prognosis 21.

HE4 has a reference value for early screening and postoperative clinical outcomes of breast cancer. The increase of HE4 indicated that the number of abnormal nuclear divisions of breast cancer cells increased and the ability of chromatin synthesis and assembly of tumor cells increased 22. High HE4 levels increased the risk of breast cancer recurrence after surgery. In postoperative studies of advanced epithelial ovarian cancer 23. Postoperative HE4 was the only statistically significant prognostic variable predicting progression-free survival. Other studies 24.

Also showed that HE4 could predict the prognosis of ovarian cancer. This study suggested that monitoring HE4 during first-line chemotherapy may help predict ovarian cancer progression and risk of recurrence.

Neu could participate in tumor proliferation and metastasis by releasing inflammatory mediators such as neutrophil elastase and interleukin-8 25. Plt could promote tumor angiogenesis and metastasis, thereby protecting tumor cells from anti-tumor immune response 26.

Therefore, high levels of Neu and Plt were conducive to tumor formation and growth and increased the possibility of breast cancer recurrence or metastasis.

A study 27, had shown that the ratio of Neu to lymphocytes affected the outcome of primary breast cancer surgery.

Another study 28, also showed that the ratio of Plt to Lym was a poor prognostic indicator of breast cancer, suggesting that Plt had a potential effect on the prognosis of breast cancer. This study did not find that Lym, NLR, and SII had a predictive effect on the prognosis of breast cancer, which was different from the results of previous studies 29, which may be related to the small sample size and different conditions of included patients.

In conclusion, serum CEA, CA153, and HE4 combined with Neu and Plt had a high value in the recurrence and metastasis of breast cancer. Therefore, in postoperative follow-up of breast cancer patients, it is necessary to detect serum tumor markers and related immune inflammatory indicators in combination, to identify patients with a high risk of postoperative recurrence and metastasis as soon as possible and then take targeted treatment. The combined detection of CEA, CA153, HE4, Neu, and Plt could improve the sensitivity and predictive efficiency of breast cancer recurrence and metastasis, which could provide a certain reference for the diagnosis of postoperative recurrence and metastasis of breast cancer. However, this study was a single-center study with a limited sample size. The correlation between CEA, CA153, HE4, Neu, Plt, and breast cancer and their predictive value for breast cancer prognosis need to be further confirmed by increasing the number of cases. In addition, future studies need to extend the follow-up time for further validation.

## Data Availability

The data used to support the findings of this study are included in the article.
